# Integrin – Dependent Mechanotransduction in Mechanically Stimulated Human Annulus Fibrosus Cells: Evidence for an Alternative Mechanotransduction Pathway Operating with Degeneration

**DOI:** 10.1371/journal.pone.0072994

**Published:** 2013-09-05

**Authors:** Hamish T. J. Gilbert, Navraj S. Nagra, Anthony J. Freemont, Sarah J. Millward-Sadler, Judith A. Hoyland

**Affiliations:** Centre for Regenerative Medicine, Institute of Inflammation and Repair, Faculty of Medical and Human Sciences, The University of Manchester, Manchester, United Kingdom; National Center for Scientific Research Demokritos, Greece

## Abstract

Intervertebral disc (IVD) cells derived from degenerate tissue respond aberrantly to mechanical stimuli, potentially due to altered mechanotransduction pathways. Elucidation of the altered, or alternative, mechanotransduction pathways operating with degeneration could yield novel targets for the treatment of IVD disease. Our aim here was to investigate the involvement of RGD-recognising integrins and associated signalling molecules in the response to cyclic tensile strain (CTS) of human annulus fibrosus (AF) cells derived from non-degenerate and degenerate IVDs. AF cells from non-degenerate and degenerate human IVDs were cyclically strained with and without function blocking RGD – peptides with 10% strain, 1.0 Hz for 20 minutes using a Flexercell® strain device. QRT-PCR and Western blotting were performed to analyse gene expression of type I collagen and ADAMTS -4, and phosphorylation of focal adhesion kinase (FAK), respectively. The response to 1.0 Hz CTS differed between the two groups of AF cells, with decreased ADAMTS -4 gene expression and decreased type I collagen gene expression post load in AF cells derived from non-degenerate and degenerate IVDs, respectively. Pre-treatment of non-degenerate AF cells with RGD peptides prevented the CTS-induced decrease in ADAMTS -4 gene expression, but caused an increase in expression at 24 hours, a response not observed in degenerate AF cells where RGD pre-treatment failed to inhibit the mechano-response. In addition, FAK phosphorylation increased in CTS stimulated AF cells derived from non-degenerate, but not degenerate IVDs, with RGD pre-treatment inhibiting the CTS – dependent increase in phosphorylated FAK. Our findings suggest that RGD -integrins are involved in the 1.0 Hz CTS – induced mechano-response observed in AF cells derived from non-degenerate, but not degenerate IVDs. This data supports our previous work, suggesting an alternative mechanotransduction pathway may be operating in degenerate AF cells.

## Introduction

The intervertebral disc (IVD) is subject to a variety of mechanical stimuli, including compressive and tensile strains, hydrostatic pressures (HP) and fluid flow [1], [2]. These forces are transduced throughout the IVD, subjecting the resident cells to mechanical stimuli, with predominantly compressive strains and HP in the nucleus pulposus (NP) and tensile strains in the annulus fibrosus (AF) [1], [2]. The effects of these mechanical stimuli on IVD cell metabolism and matrix regulation are now being defined, with the type, magnitude, frequency and duration of force, all important factors in determining how disc cells respond [3], [4], [5], [6], [7], [8], [9], [10], [11], [12]. Furthermore, physiological mechanical stimuli have been shown to lead to matrix anabolism in healthy disc cells [9], [11], [12], [13], [14], [15], [16], [17], while non-physiological mechanical stimuli (i.e. overloading and immobilisation) lead to matrix catabolism [5], [8], [9], [12], [18], [19].

Degenerative disc disease (DDD), characterised by the degradation of IVD matrix, has been shown to influence the mechano-response of disc cells, with cells derived from degenerate IVDs responding aberrantly to mechanical stimuli compared to cells from non-degenerate IVDs. Le Maitre *et al.* reported a lack of response to HP in human NP cells derived from degenerate IVDs, in contrast to the anabolic response observed in human NP cells derived from non-degenerate IVDs [20]. Furthermore, we have recently shown that the reduction in catabolic gene expression observed in human AF cells derived from non-degenerate IVDs subjected to a physiologically relevant stimulus of 1.0 Hz cyclic tensile strain (CTS), is replaced by a reduction in anabolic gene expression in cells derived from a degenerate disc [12]. Such findings suggest that physiological mechanical stimuli important for matrix homeostasis could, in fact, become detrimental with IVD degeneration resulting in aberrant cell responses and potentially leading to progression of DDD. It is therefore important to investigate the molecular mechanisms involved in disc cell mechanotransduction. Importantly, if the mechanotransduction pathways are found to be altered in disc cells derived from degenerate tissue, then elucidation of the pathways could lead to an improved understanding of the aetiology of DDD and potentially to the discovery of novel therapeutic targets for the prevention, and/or treatment, of IVD degeneration.

To date, studies investigating the mechanotransduction pathways operating in disc cells are limited. Li *et al*. recently reported remodelling of the actin cytoskeleton in both outer AF and NP cells following treatment with CTS [21] and we recently reported the involvement of interleukin (IL) -1 and IL -4 in the 1.0 Hz CTS-induced reduction in catabolic gene expression in human AF cells derived from non-degenerate IVDs [22]. Interestingly, we observed that this cytokine – dependent mechano-response was exclusive to AF cells derived from non-degenerate tissue, with the decrease in anabolic gene expression observed in 1.0 Hz CTS – stimulated degenerate AF cells occurring independently of either cytokine [22]. Such findings suggest that the aberrant mechano-responses observed in disc cells derived from degenerate tissue occur through an altered or different mechanotransduction pathway.

Further upstream in the mechanotransduction pathway, prior to both soluble mediator release and intracellular signalling, is the process of cellular mechano-sensing. Mechano-sensing is the process whereby a cell is able to sense a mechanical stimulus and convert this physical stimulus into a biochemical signal. Integrins, a family of heterodimeric transmembrane glyco-proteins comprised of an α and β subunit [23], are perfectly poised to function as transducers of mechanical stimuli. Previous studies have reported a role for integrins as mechano-receptors in various cell types, including cardiovascular cells [24], bone cells [25], chondrocytes [26], [27], [28], [29] and IVD cells [30]. Following integrin activation, clustering of the integrin receptors occurs with recruitment of adapter proteins and non-receptor kinases to the cytoplasmic tail of the β subunit (see review by Liu *et al*. [31]). Focal adhesion kinase (FAK), recruited by the integrin-associated adapter proteins, appears to play a central role in the processing of intracellular signals via integrin activation and has been shown to be phosphorylated following mechanical stimulation in a number of cells [32], [33], [34], including chondrocytes [35].

IVD cells have been shown to express many of the integrin subunits expressed by chondrocytes [36], [37], [38] and a role for integrins in the mechano-response of IVD cells has been suggested [30].

Recently, Le Maitre and colleagues reported that the HP – induced decrease in aggrecan gene expression observed in human NP cells could be inhibited by pre-treatment with RGD – function blocking peptides in NP cells derived from non-degenerate, but not degenerate IVDs [30]. Such findings not only implicate RGD – recognising integrins in the mechano-response of NP cells exposed to HP, but also suggest that an alternative mechanotransduction pathway may be operating in NP cells in degeneration. To date, however, the involvement of integrins in the mechanotransduction pathway of human AF cells has not been investigated. Therefore, the aims of this study were: i) to investigate the involvement of integrins, specifically RGD – recognising integrins, in the mechano-response of human AF cells following treatment with 1.0 Hz CTS; ii) to investigate the activation of FAK via tyrosine 397 phosphorylation in human AF cells exposed to 1.0 Hz CTS and to determine whether FAK activation was integrin-dependent; and iii) to ascertain whether the previously reported altered mechanotransduction pathway operating in AF cells derived from degenerate tissue [22] was due to altered integrin involvement.

## Materials and Methods

### Ethics Statement

All human tissue was collected and used with patient or relatives’ written consent and approval from both the North West Research Ethics Committee (08/H1010/36) and the University of Manchester Research Ethics Committee.

### IVD Tissue

Human IVD tissue was collected from patients undergoing lumbar spinal surgery for degenerative disc disease (DDD) or from cadavers (within 18 hours of death). Tissue was processed for cell extraction and representative samples of all tissues containing intact AF and NP regions were formalin-fixed, paraffin-embedded and sections histologically graded as previously reported [12]. Patient tissue samples (n = 6) were used for the gene expression analysis of mechanically stimulated AF cells, with 3 patient samples classed as non-degenerate (mean donor age 47 years, age range 37–57 years) and 3 patient samples classed as degenerate (mean donor age 41 years, age range 29–49 years). For protein analysis of mechanically stimulated AF cells, 4 patient tissue samples (classed as non-degenerate; mean donor age 50 years, age range 36–63 years) and 3 patient tissue samples (classed as degenerate; mean donor age 43 years, age range 42–46 years) were used.

### Isolation and Culture of AF Cells

AF tissue was separated from the IVD and finely minced prior to enzymatic digestion as previously reported [12]. AF cells were cultured in standard medium (Dulbecco’s modified Eagle’s medium with glucose 4.5 g/L, Glutamax™ and pyruvate (Gibco) containing 50 µg/mL ascorbic acid, 250 ng/mL amphotericin, 100 U/mL penicillin, 100 µg/mL streptomycin (Invitrogen) and 10% (v/v) foetal calf serum (Invitrogen)) and expanded in monolayer. Subconfluent AF cells (passage numbers of ≤6) were trypsinised (Invitrogen) and seeded into untreated silicone membrane BioFlex® 6 well culture plates (Flexcell International) at a density of 2×10^5^ cells/well or 5×10^5^ cells/well (for mRNA or protein analysis, respectively) and allowed to adhere for 48 hours. Media was changed to serum-free media 15 hours prior to the application of CTS.

### Application of CTS in the Presence and Absence of RGD Peptides

AF cells in serum-free media adhered to Bioflex® culture plates were treated with or without RAD control peptides (50 µg/mL) (Calbiochem, Cat no. 03-34-0052) or the function blocking peptides RGD (50 µg/mL) (Calbiochem, Cat no. 03-34-0035) 30 minutes prior to the application of CTS (peptide concentrations and treatment durations obtained from previous chondrocyte and NP cell studies [30], [39]). A CTS of 10% strain at 1.0 Hz frequency for 20 minutes, was delivered to the base of the silicone membranes within the Bioflex**®** culture plates using the FX-4000™ Flexercell® Tension™ System (Flexcell International), and consequently to AF cells adhered to these membranes, using computer controlled negative pressure, as previously described in detail [12]. Unstimulated AF cells adhered to Bioflex® plates served as controls. Mechanically stimulated and unstimulated peptide-treated and untreated AF cells were incubated at 37°C with 5% CO_2_ for either 0 (baseline control), 1, 3 or 24 hours and total RNA extracted. Timepoints for mRNA analysis were chosen based on previously published observations, where gene expression was altered at 1 and 24 hours in AF cells derived from non-degenerate and degenerate IVDs, respectively [12].

For protein analysis, mechanically stimulated (10% strain, 1.0 Hz frequency) and unstimulated AF cells derived from non-degenerate and degenerate IVDs, in the absence of peptides, were lysed immediately following the completion of 5, 10 or 20 minute durations of CTS. Having established that activation of FAK was greatest after 20 minutes of CTS, non-degenerate AF cells were then subsequently mechanically stimulated (10% strain, 1.0 Hz frequency) for 5 minutes (to enable comparison with a time point shown to have baseline phosphorylated FAK levels) and 20 minutes, in the presence and absence of RAD (50 µg/mL) or RGD (50 µg/mL) peptides and total protein collected following immediate cell lysis.

### Cell Viability

Cell viability was assessed pre- and post- CTS using the trypan blue (0.4%) (Sigma) exclusion assay as previously reported [12].

### Quantitative Real Time PCR (QRT-PCR)

Total RNA was extracted from each BioFlex® culture plate well using TRIzol™ (Invitrogen) according to the manufacturer’s instructions and samples treated with DNase I (Ambion) as previously reported [12]. RNA quality and quantity were determined using the Nanodrop ND-1000 Spectrophotometer (Nanodrop Technologies) and 500 ng of RNA reverse transcribed using the High Capacity Reverse Transcription Kit (Applied Biosystems). QRT-PCR was performed in triplicate using TaqMan® Universal PCR Master Mix (Applied Biosystems) with primers and probes for glyceraldehyde 3-phosphate dehydrogenase (GAPDH), type I collagen and a disintegrin and metalloproteinase with a thrombospondin type 1 motif (ADAMTS) -4, using previously published sequences and concentrations [12]. Data was analysed using the 2-ΔΔCt method and normalised to the endogenous control gene GAPDH and unloaded baseline controls [40].

### Western Blot Analysis

Total protein was extracted using radioimmunoprecipitation assay (RIPA) lysis buffer (50 mM Tris-HCL, 1% Triton-X (Sigma), 0.25% Sodium deoxycholate (Sigma), 150 mM NaCl (Sigma), Halt™ Protease/Phosphatase inhibitor Single-Use cocktail (Thermo Scientific)) at 4°C with frequent agitation for 20 minutes. Cell lysates were cleared of insoluble debris by centrifugation at 12,000×g for 10 minutes at 4°C and protein quantified using the Pierce® BCA Protein Assay according to the manufacturer’s instructions. Whole cell lysates were reduced by incubation with 5X Laemmli reducing buffer (250 mM Tris-HCl (pH 6.8), 25% Glycerol (Sigma), 10% sodium dodecyl sulphate (SDS), 500 mM Dithiothreitol (DTT) (Sigma), 0.05% Bromophenol blue dye (Sigma)) at 95°C for 10 minutes. Equal amounts of cell lysates (5 µg/well) were separated using 10% SDS – polyacrylamide gel electrophoresis (SDS – PAGE) and protein transferred to polyvinylidene fluoride (PVDF) membranes (GE Healthcare). PVDF membranes were first blocked with 5% (w/v) bovine serum albumin (BSA) (Sigma) in Tris-buffered saline (50 mM Tris, 139 mM NaCl, pH 7.6) containing 0.1% Tween 20 (Sigma) (TBS-T) overnight at 4°C with constant agitation. Membranes were then washed with TBS-T and incubated with 2% (w/v) BSA in TBS-T with anti – phosphorylated FAK antibody (R&D Systems; MAB4467) for 2 hours at room temperature with constant agitation. Membranes were washed 3 times with TBS-T and incubated with 2% (w/v) BSA in TBS-T with horseradish peroxidise-conjugated secondary antibody (R&D Systems; HAF008) for 1 hour at room temperature. Membranes were washed 3 times with TBS-T, developed using ECL chemiluminescent reagent (PerkinElmer) according to the manufacturer’s protocol and exposed to photographic film (Blue XB, Kodak). Membranes were then incubated with stripping buffer (50 mM glycine, 10% w/v SDS) at 55°C with constant agitation for 30 minutes to remove secondary antibody, the membranes washed 3 times in TBS-T and incubated with 5% (w/v) non-fat milk (Alcafe) in TBS-T overnight at 4°C. Following 3 washes with TBS-T, the membranes were incubated with 2% (w/v) non-fat milk in TBS-T with anti – total FAK antibody (R&D Systems; MAB4467) for 2 hours at room temperature with constant agitation. Membranes were then washed 3 times with TBS-T and then incubated with 2% (w/v) non-fat milk in TBS-T with horseradish peroxidise-conjugated secondary antibody (R&D Systems; HAF018) for 1 hour at room temperature and protein visualised as described above. The density of each protein band was quantified using a Syngene imaging system, and the ratio of phosphorylated to total protein was calculated, normalised to unstimulated timepoint controls and the mean of the patient samples plotted as % change.

### Statistics

Statistical analysis was performed using the non-parametric Mann-Whitney U test, with *p* values of ≤0.05 reported as significant.

## Results

### Cell Viability

AF cells isolated from non-degenerate and degenerate IVDs remained viable (>90%) throughout the culture period, with cell viability unaffected by mechanical stimulation or peptide treatment. AF cell morphology remained fibroblastic-like throughout the culture period, with and without peptide treatment and/or mechanical stimulation.

### Gene Expression Analysis

The genes chosen for analysis were based on previously published observations, whereby from a panel of extracellular matrix and matrix degrading enzyme genes investigated, only MMP -3 and ADAMTS -4, and aggrecan and type I collagen gene expression were altered in 1.0 Hz CTS treated non-degenerate and degenerate AF cells, respectively [40]. ADAMTS -4 and type I collagen were chosen as the genes to be analysed, as these genes had shown the greatest fold changes in response to 1.0 Hz CTS of non-degenerate and degenerate AF cells, respectively [22].

Importantly, RAD or RGD –peptide treatment in the absence of mechanical stimulation had no effect on the baseline gene expression of any of the genes analysed. Unloaded controls showed no significant change in gene expression for the genes investigated at any of the time points analysed (data not shown).

### Mechanical Stimulation (1.0 Hz CTS) of Peptide Treated AF Cells

#### Non-degenerate AF cells

**Figure 1:**
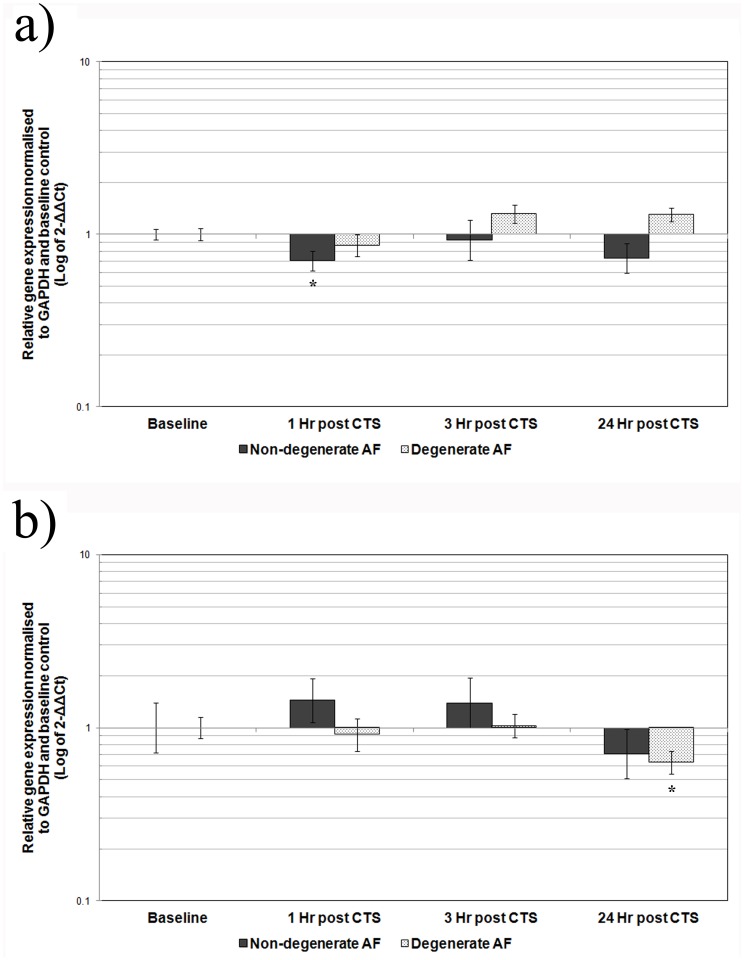
Effect of CTS on gene expression in AF cells derived from non-degenerate and degenerate IVDs. Cells derived from non-degenerate (n = 3) or degenerate (n = 3) IVDs were mechanically stimulated with 10% CTS, 1.0 Hz frequency for 20 minutes and then incubated for up to 24 hours prior to analysis. QRT-PCR was used to analyse the gene expression of **A**) ADAMTS -4 or **B**) type I collagen, relative to the housekeeping gene GAPDH and normalised to the corresponding unloaded baseline control. Black and grey represent mechanically stimulated non-degenerate and degenerate AF cells, respectively. Values are mean of 3 donors+/− standard error mean. * denote a significant change (*p*≤0.05) in gene expression between mechanically stimulated and unstimulated baseline control.

**Figure 2:**
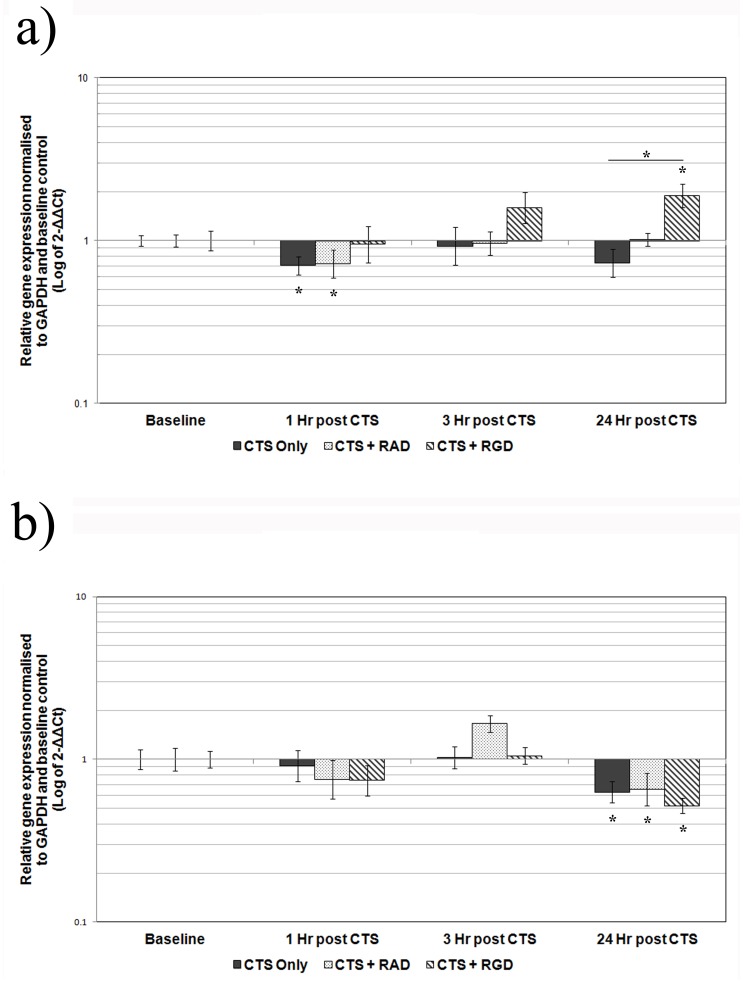
Effect of CTS on the gene expression of AF cells from non-degenerate and degenerate IVDs+/− peptides. Cells derived from non-degenerate and degenerate IVDs were treated+/− RAD (50 µg/ml) or RGD (50 µg/ml) -peptides 30 minutes prior to mechanical stimulation with CTS at 10% strain, 1.0 Hz, for 20 minutes, then incubated for up to 24 hours prior to analysis. QRT-PCR was used to analyse the gene expression of A) ADAMTS -4 or B) type I collagen, relative to the housekeeping gene GAPDH and normalised to the corresponding unloaded baseline control in non-degenerate (n = 3) and degenerate (n = 3) AF cells, respectively. Black represents AF cells cyclically strained without peptide treatment, while speckles and stripes represent cells cyclically strained after treatment with RAD or RGD –peptides, respectively. Values are mean of 3 donors+/− standard error mean. *denote a significant change (*p*≤0.05) in gene expression between mechanically stimulated and unstimulated baseline control.

There was a significant decrease in the relative gene expression of ADAMTS -4 at 1 hour post application of 1.0 Hz CTS compared to baseline (4 fold, *p*≤0.05), similar to that previously reported [12], with expression levels returning to baseline by 3 hours (Figure 1A). Treatment of the non-degenerate AF cells with RAD –peptides prior to the application of CTS had no effect on the CTS – induced decrease in ADAMTS -4 gene expression, which remained significantly decreased compared to baseline (*p*≤0.05) (Figure 2A). However, pre-treatment of non-degenerate AF cells with the function blocking RGD –peptides prevented the CTS – induced decrease in ADAMTS -4 gene expression at 1 hour post-CTS (Figure 2A). Furthermore, pre-treatment of non-degenerate AF cells with RGD peptides resulted in a significant (*p*≤0.05) increase in ADAMTS -4 gene expression (2 fold) at 24 hours post-CTS, compared to baseline and to non-degenerate AF cells mechanically stimulated in the absence of peptides (Figure 2A). There was no change in the relative gene expression of type I collagen at any time point following 1.0 Hz CTS treatment of non-degenerate AF cells, as previously reported [12] (Figure 1B).

#### Degenerate AF cells

The relative gene expression of ADAMTS -4 remained unchanged at all time points following 1.0 Hz CTS stimulation of degenerate AF cells, as previously reported [12] (Figure 1A). However, the relative gene expression of type I collagen was significantly decreased compared to baseline at 24 hours post 1.0 Hz CTS (4 fold, *p*≤0.05), consistent with that previously reported [12] (Figure 1B). Pre-treatment of degenerate AF cells with either RAD or RGD –peptides had no effect on the CTS – induced decrease in type I collagen gene expression, which remained decreased compared to baseline (4 fold, *p*≤0.05 and 6 fold, *p*≤0.05, respectively) (Figure 2B).

### CTS – Induced Activation of Focal Adhesion Kinase (FAK) in Non-degenerate, but not Degenerate AF Cells

**Figure 3:**
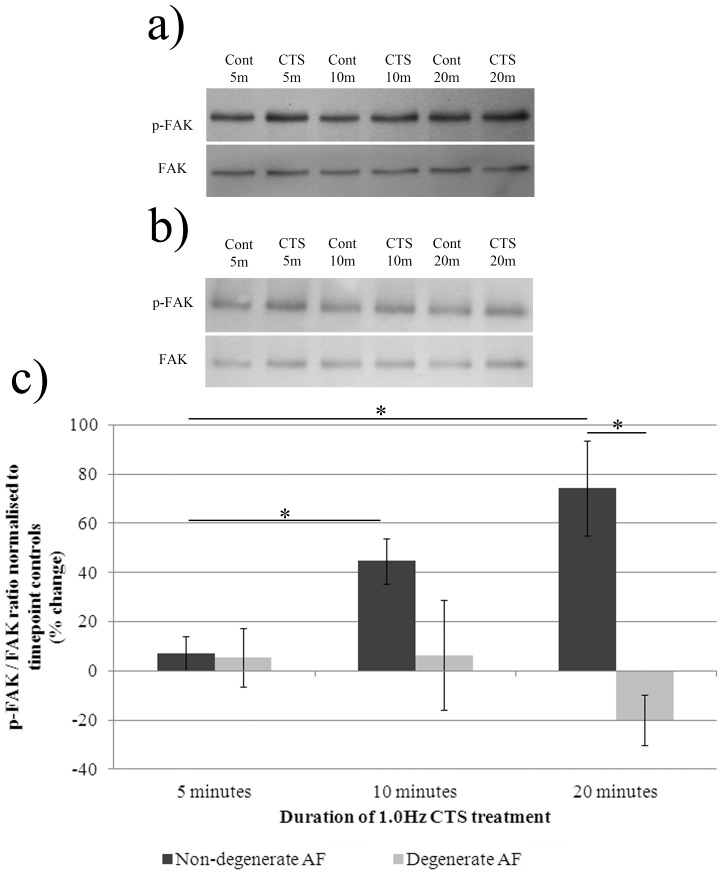
Phosphorylation of FAK following treatment of AF cells derived from non-degenerate and degenerate IVDs with 1.0 Hz CTS. AF cells derived from non-degenerate (n = 4) and degenerate (n = 3) IVDs were treated+/−1.0 Hz CTS in serum-free media and total protein extracted at timepoints of up to 20 minutes. Mechanically stimulated and unstimulated A) non-degenerate and B) degenerate protein samples (5 µg/well) were separated using 10% SDS-PAGE and probed using primary antibodies against phosphorylated FAK. Blots were then stripped using a stripping buffer, re-blocked and probed using an antibody against total FAK protein. C) The density of bands was quantified using a Syngene imaging system and the ratio of phosphorylated: total FAK protein normalised to timepoint controls and plotted as % change. *denotes a significant change (*p*≤0.05) between treatment groups.

Following application of 1.0 Hz CTS, non-degenerate AF cells showed a significant increase (45% increase) in phosphorylated FAK (tyrosine 397) relative to total FAK protein at 10 minutes compared to 5 minutes CTS (*p*≤0.05) (Figure 3). The levels of phosphorylated FAK increased further following 20 minutes of CTS (74% increase when compared to 5 minutes CTS (*p*≤0.05)) (Figure 3A and C). In the CTS treated degenerate AF cells levels of phosphorylated FAK (tyr397) remained unchanged compared to total FAK at all three time points (Figure 3B and C).

### CTS – Induced Activation of FAK in Non-degenerate AF Cells is RGD – integrin Dependent

**Figure 4:**
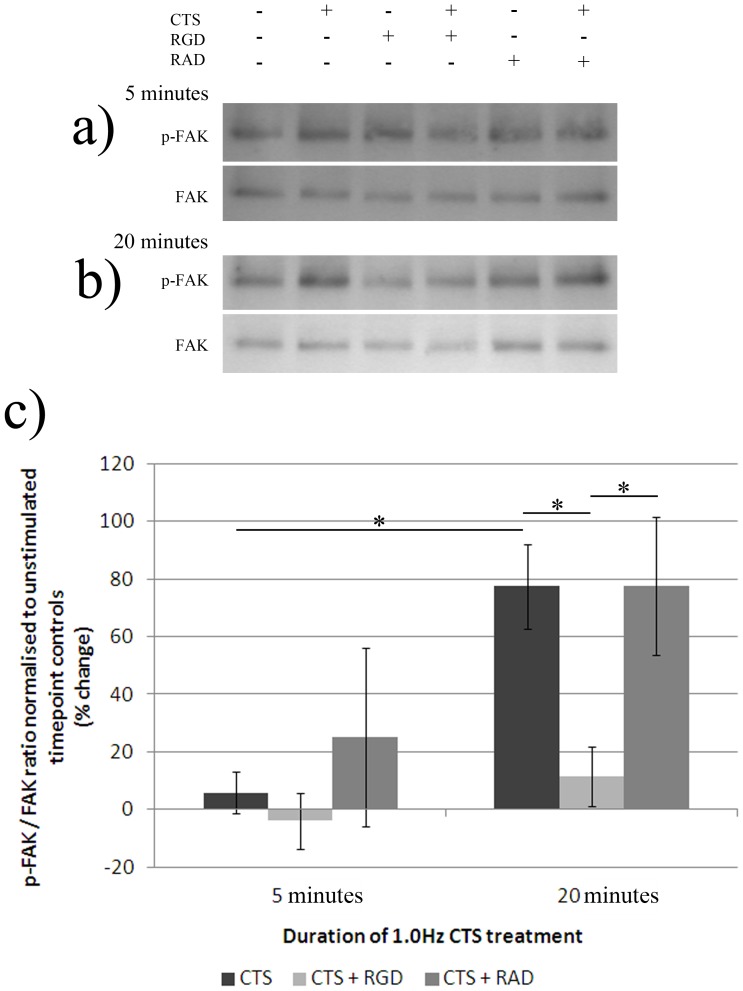
Phosphorylation of FAK following treatment of AF cells derived from non-degenerate IVDs with 1.0 Hz CTS, with and without pre-treatment with RGD or RAD peptides. AF cells derived from non-degenerate IVDs (n = 4) were treated+/− RGD (50 µg/ml) or RAD (50 µg/ml) peptides, and mechanically stimulated (10% CTS, 1.0 Hz frequency) in serum-free media and total protein extracted at timepoints of 5 and 20 minutes. Mechanically stimulated and unstimulated,+/− RGD or RAD peptides, non-degenerate protein samples (5 µg/well) exposed to A) 5 minutes and B) 20 minutes of CTS, were separated using 10% SDS-PAGE and probed using primary antibodies against phosphorylated FAK. Blots were then stripped using a stripping buffer, re-blocked and probed using an antibody against total FAK protein. C) The density of bands were quantified using a Syngene imaging system and the ratio of phosphorylated: total FAK protein normalised to timepoint controls and plotted as % change. *denotes a significant change (*p*≤0.05) between treatment groups.

Mechanical stimulation of non-degenerate AF cells with 20 minutes CTS (10% strain, 1.0 Hz) led to a significant increase in phosphorylated FAK (tyr397), similar to that reported above (77% increase+/−15%) in FAK phosphorylation versus total FAK protein, when compared to levels at 5 minutes CTS (*p*≤0.05) (Figure 4). Pre-treatment with RGD, inhibited the CTS – induced increase in FAK phosphorylation (tyr397), with levels of phosphorylated FAK remaining significantly lower than cells exposed to CTS with and without RAD peptide pre-treatment (*p*≤0.05) (Figure 4). Mechanically stimulated non-degenerate AF cells pre-treated with RAD peptides demonstrated increased FAK phosphorylation (tyr397) at the 20 minute timepoint to a similar level as that observed with the CTS only treated cells (77% increase+/−24%), with no significant difference between CTS only, or CTS plus RAD peptide pre-treatment groups (Figure 4).

## Discussion

Mechanical stimuli influence AF cell metabolism and function, with physiologically relevant magnitudes, frequencies and durations of force regulating extracellular matrix (ECM) homeostasis. Recently, the mechanobiology of AF cells has been shown to be altered with disc degeneration (resulting in reduced matrix protein gene expression): an aberrant response that appears to occur through an altered mechanotransduction pathway (loss of IL -1 and -4 involvement) [22]. To date, the involvement of integrins as mechano-receptors has been investigated in a variety of cell types [24], [25], [26], [27], [28], [29], [41], including NP cells (exposed to their predominant mechanical stimulus, cyclic compression) [30], but has not, until now, been investigated in AF cells.

Here we report, in agreement with our previous study [12], that the mechano-response of human AF cells to 1.0 Hz CTS differs between AF cells derived from non-degenerate and degenerate IVDs, with ADAMTS -4 gene expression decreased in non-degenerate AF cells and type I collagen gene expression decreased in degenerate AF cells. This difference in CTS-stimulated gene expression between AF cells of non-degenerate and degenerate origin illustrates a difference in the mechano-response and appears not to be due to a difference in response time as there was no change in type I collagen or ADAMTS -4 gene expression in non-degenerate or degenerate AF cells respectively, at any of the time points analysed. Although we did not investigate the mechano-response occurring beyond 24 hours, findings presented by MacLean *et al.* suggest that changes in IVD gene expression of matrix proteins and matrix degrading enzymes (including type I collagen and ADAMTS -4, as investigated here) following a single loading event, can be observed across all genes, up to 24 hour post load. Interestingly, the authors found that including a timepoint of 72 hours post load did not highlight *de novo* changes in gene expression for any genes which had not already shown a significant change in expression by 24 hours [42]. Therefore, studying effects at 24 hours post load was deemed sufficient to investigate the mechano-response of AF cells stimulated with a single CTS stimulation.

In order to ascertain whether integrins were involved in this mechano-response, function blocking peptides were used during the loading regime. Importantly, although previous studies have reported high levels of cell death due to apoptosis following RGD peptide treatment of cells [43], [44], [45], [46], treatment of AF cells with either RAD control peptides or RGD function blocking peptides had no effect on cell viability. The high levels of cell viability (>90%), along with observations of normal and consistent cell morphology up to 24 hours post peptide treatment, suggests that the cells were not apoptotic. Furthermore, treatment of AF cells with either RAD control peptides or function blocking RGD peptides had no effect on the baseline gene expression levels of ADAMTS -4 or type I collagen in AF cells derived from non-degenerate and degenerate IVDs respectively, suggesting that basal gene expression for these two genes occurs independently of RGD – recognising integrins. This is in agreement with other studies which have used function blocking RGD peptides as inhibitory molecules for the elucidation of integrin mechanotransduction pathways where no effect on basal gene expression or resting membrane potential was reported [29], [39], [47].

Importantly, the data presented here illustrates the involvement of RGD–recognising integrins in the mechano-response of human AF cells derived from non-degenerate IVDs exposed to 1.0 Hz CTS. Furthermore, we show that RGD – recognising integrins do not appear to be involved in the altered mechano-response (i.e. no change in ADAMTS -4 but decreased type I collagen gene expression) of human AF cells derived from degenerate IVDs exposed to an identical stimulus. This suggests that the aberrant response in degenerate cells is recognised by other mechano-receptors and occurs through an alternative mechanotransduction pathway.

Le Maitre *et al.* also found that mechanical compression induced decreases in aggrecan gene expression could be prevented by RGD peptide pre-treatment of NP cells derived from non-degenerate, but not degenerate IVDs, with the classic fibronectin receptor α5β1 being proposed as the integrin involved [30]. Taken together, this study and that by Le Maitre and colleagues suggest a role for RGD-integrins as mechano-receptors in mechanically stimulated AF and NP cells. Interestingly however, involvement of these RGD-integrins in both cell types appears to then be lost with degeneration, suggesting that RGD integrin mediated mechanotransduction may not be operational in cells derived from degenerate IVDs, although they have been shown to express integrin subunits [36], [37], [38].

Pre-treatment with RGD peptides of non-degenerate AF cells prior to mechanical stimulation not only prevented the CTS-induced decrease in ADAMTS -4 gene expression observed at the 1 hour time point, but led to a subsequent increase in ADAMTS -4 gene expression. These findings suggest that by preventing the involvement of RGD-recognising integrins during non-degenerate AF cell mechanotransduction, alternative mechano-receptors are utilised, leading to an altered and delayed mechano-response following 1.0 Hz CTS. Potential alternative mechano-receptors include the non-RGD integrins (e.g. the collagen receptors α1β1 and α2β1), as well as the heparin sulphate proteoglycans syndecans [48] and mechano-sensitive ion channels (see review by Sachs [49]).

Having ascertained involvement of RGD – recognising integrins in the mechano-response of AF cells derived from non-degenerate IVDs, the CTS – induced activation of FAK, an integrin-associated non-receptor tyrosine kinase was studied, by analysing the phosphorylation of tyrosine 397. FAK is an integrin-associated tyrosine kinase which becomes phosphorylated at tyrosine 397 upon integrin activation. FAK activity has been shown to increase following treatment of a number of cell types with CTS [32], [33], [34], [35]. Although the activity of FAK has never been investigated in IVD cells, its involvement in the integrin – dependent mechanotransduction pathway of chondrocytes has been reported. Lee *et al*. found that, upon CTS stimulation of chondrocytes derived from both non-osteoarthritic and osteoarthritic cartilage, there was rapid tyrosine phosphorylation (within 1 minute) of FAK, paxillin and β-catenin, which could be inhibited following pre-treatment of cells with RGD peptides [35]. In our study, levels of phosphorylated FAK increased following CTS treatment in AF cells derived from non-degenerate tissue. It appears therefore that, as with chondrocytes, AF cells derived from non-degenerate, but not degenerate IVDs, respond to CTS by increasing FAK phosphorylation; however, the duration of mechanical stimulus needed to elicit a response is much greater. This difference in the CTS – induced activation time of FAK could be due to differences in the mechano-biology between AF cells and chondrocytes, or differences between the mechanical stimuli applied.

Similarly to that reported in chondrocytes by Lee *et al.* [35], pre-treatment with RGD function blocking peptides was able to inhibit the CTS – induced increase in FAK phosphorylation in non-degenerate AF cells, suggesting that CTS causes activation of FAK in an RGD-integrin dependent manner. Although it is beyond the scope of this manuscript to directly link the activation of FAK with changes in gene expression, the evidence reported here suggests that the decrease in ADAMTS -4 gene expression observed in CTS stimulated non-degenerate AF cells occurs through an integrin, and potentially FAK, -dependent pathway.

Interestingly, AF cells derived from degenerate tissue failed to respond to CTS in a similar manner to AF cells derived from non-degenerate tissue, with levels of phosphorylated FAK (tyr397) remaining at baseline values. The lack of FAK activation observed in CTS stimulated degenerate AF cells suggests involvement of alternative mechano-receptors and it would therefore be of interest to investigate the involvement of other non-RGD recognising receptors (e.g. the collagen receptors α1β1 and α2β1 integrins (see review by Loeser [37]), and the hyaluronan receptor CD44 [50]) in AF cells derived from degenerate IVDs.

In conclusion, we have shown that the mechano-response of AF cells derived from non-degenerate IVDs exposed to 10% strain, 1.0 Hz frequency for 20 minutes, appears to occur in an RGD – recognising integrin – dependent manner and that exposure of these cells to this mechanical stimulus leads to integrin – dependent phosphorylation of FAK. Furthermore, the altered mechano-response observed in AF cells derived from degenerate IVDs exposed to an identical stimulus, appears to occur independently of RGD – recognising integrins, with no increase in FAK phosphorylation. These findings, supported by that reported by Le Maitre *et al*. [30], suggest that cells derived from non-degenerate IVDs utilise RGD-integrins in their mechano-response, with FAK activation likely to be involved. Interestingly, RGD-integrins and FAK appear not to be involved in the mechano-response of degenerate AF cells. Further studies are needed to further elucidate other mechanotransduction pathways operating in IVD cells from both non-degenerate and degenerate tissue, as identifying discrepancies occurring with degeneration could lead to the discovery of novel therapeutic targets for the prevention and/or treatment of DDD.
